# Assessment of successful percutaneous mitral commissurotomy by MRproANP and sCD146

**DOI:** 10.1186/s12872-020-01435-y

**Published:** 2020-04-05

**Authors:** Marc Badoz, Mattia Arrigo, Anne-Claire Mogenet, Malha Sadoune, Nicolas Meneveau, Alexandre Mebazaa, Marie-France Seronde

**Affiliations:** 1 Besancon University Hospital, and EA3920, University of Burgundy Franche-Comté, Besancon, France; 2 INSERM UMR-S 942, Paris, France; 3 Department of Anesthesiology and Critical Care Medicine, AP-HP, Saint Louis Lariboisière University Hospitals, Paris, France; 4 Department of Cardiology, AP-HP, Saint Louis Lariboisière University Hospitals, Paris, France; 5 Department of Cardiology, University Heart Center, University Hospital Zurich, Zurich, Switzerland

**Keywords:** Mitral stenosis, Biomarker, Brain natriuretic peptide, MRproANP

## Abstract

**Background:**

We studied the course of plasma concentrations of 4 cardiovascular biomarkers: natriuretic peptides (BNP, NT-proBNP; mid-regional (MR) pro-atrial NP); and soluble endothelial CD146 (sCD146), in patients with severe mitral valve stenosis undergoing percutaneous mitral commissurotomy (PMC) to identify potential markers of procedural success.

**Methods:**

Biomarkers were tested in 40 patients the day before and the day after PMC. Success was defined as mitral valve area ≥ 1.5 cm^2^; or an increase of ≥0.5 cm2 in mitral valve area associated with echocardiographic mitral regurgitation <grade 3–4 post-PMC.

**Results:**

Average age was 63.5 ± 12.7 years; 32(80%) were female. Before PMC, mean valve area was 1.1 ± 0.2 cm^2^, mean gradient 9.1 ± 3.5 mmHg. PMC was successful in 30 (75%) and unsuccessful in 10 (25%). PMC yielded a significant reduction in MR-proANP and sCD146, driven by a significant reduction in these biomarkers in patients with successful procedure, whereas no reduction was observed in patients with unsuccessful procedure. A significant correlation was found between changes in plasma sCD146 and the relative change in mitral valve area. Elevated pre-procedural sPAP correlated with high sCD146, and accordingly, a significant correlation between the decrease in sPAP and sCD146 after PMC was shown.

**Conclusions:**

MR-proANP and plasma sCD146 decreased significantly immediately after successful PMC. They appear to be markers of immediate success of PMC and of the hemodynamic improvement achieved by this procedure in patients with MS.

**Trial registration:**

This study is part of the cohorts registered with ClinicalTrials.gov on June 16, 2011 under the number NCT01374880.

## Background

The use of natriuretic peptides has become widespread in cardiology, with brain natriuretic peptide (BNP) and N-terminal pro-B-type natriuretic peptide (NT-proBNP) used daily as markers of heart failure [1–3]. However, these two biomarkers remain imperfect for the diagnosis of pulmonary and systemic congestion [4–6]. Therefore, combining BNP/NT-proBNP with other biomarkers could help to improve their diagnostic value. In this regard, other natriuretic peptides such as mid-regional pro-atrial natriuretic peptide (MR-proANP) are emerging as biomarkers that are more strongly associated with venous congestion of cardiac origin [7, 8].

Furthermore, the soluble form of CD146 (sCD146) - an adhesion molecule expressed by vascular endothelial cells and implicated in vessel integrity - is released by the endothelium [9] and circulating sCD146 levels are related to pulmonary congestion in heart failure [10, 11]. However, the physiology of sCD146 release remains poorly described. Some authors have shown that sCD146 is released from the peripheral veins in response to stretch [12], but the involvement of the heart cavities, notably the left ventricle, and pulmonary circulation in the release of sCD146 during congestion is unknown.

Mitral stenosis (MS) is a clinical condition that induces left atrial and pulmonary congestion, with preservation of the left ventricle. Successful percutaneous mitral commissurotomy (PMC) reduces left atrial and pulmonary pressures. In this context, the objectives of the present study were 1) to describe the behaviour of circulating natriuretic peptides, namely BNP, NT-proBNP, MR-proANP, and sCD146 before and after PMC for severe MS, and 2) to investigate a potential role of these cardiovascular biomarkers to assess procedural success of PMC.

## Methods

Prospective study performed in two university hospitals (University Hospital Jean Minjoz, Besancon, and University Hospital Bichat, Paris, France). Biomarker measurements were performed in the UMR-S942 Inserm, Paris, France. The study was approved by the Institutional Review Board of Paris North Hospitals, Paris 7 University, AP-HP, Paris, France (Comité d’Evaluation de l’Ethique des Projets de Recherche Biomédicale (CEERB) du GHU Nord, IRB N°00006477) under the number 10–017. All patients provided written informed consent.

### Study population

Patients with symptomatic severe rheumatic MS with an indication for PMC were included.

### Echocardiographic evaluation

All patients underwent transthoracic (TTE) and trans-oesophageal echocardiography (TOE) by trained operators on the day before PMC. Echocardiographic control by TTE was performed 24 h after the procedure to evaluate procedural success.

Echocardiography was performed to assess left ventricular ejection fraction (LVEF), measured using Simpson’s biplane method, severity of MS as assessed by the mean transmitral gradient on continuous transmitral Doppler, and mitral valve area as assessed by planimetry in the parasternal short axis view in accordance with current guidelines (14). MS was considered severe if the valve area was ≤1.5 cm^2^ by planimetry. Further, the anatomy of the mitral valve was classified according to the Wilkins score [13], which takes into account leaflet mobility, leaflet thickening, subvalvular thickening, and calcification. A Wilkins’ score > 8 is associated with advanced leaflet deformity and unfavorable anatomy, while a score ≤ 8 corresponds to a valve anatomy more amenable to PMC. Mitral regurgitation grade was evaluated semi-quantitatively by color flow Doppler imaging [14]. Left atrial diameter was measured in the parasternal long axis view; left atrial area and volume were evaluated in the 2-chamber and 4-chamber apical views. The left atrium was considered to be dilated if diameter > 40 mm or area > 20 cm^2^, or volume > 60 mL. Systolic pulmonary artery pressure was calculated according to usual techniques and a diagnosis of pulmonary arterial hypertension was retained if peak tricuspid regurgitant velocity > 2.8 m/sec [15]. Significant (moderate or severe) aortic stenosis was defined as a mean transaortic gradient > 20 mmHg by transthoracic echocardiography.

#### Definition of procedural success of PMC

Successful PMC was defined as a mitral valve area ≥ 1.5 cm^2^; or as an increase of ≥0.5 cm^2^ in mitral valve area associated with mitral regurgitation less than grade 3–4 post-PMC as measured by echocardiography. Unsuccessful procedural outcome was defined as a post-PMC mitral valve area < 1.5 cm^2^; or an increase of < 0.5 cm^2^ in mitral valve area or appearance of mitral regurgitation ≥ grade 3–4 [16].

### PMC procedure

All procedures were performed in the cardiac catheterization laboratory under local anesthetic by trained, experienced operators using the femoral approach. The Inoue balloon (Toray Medical Co. Ltd., Tokyo, Japan) was used in all procedures. The balloon was inflated gradually with echocardiographic guidance after each inflation to check for appearance or aggravation of mitral regurgitation and opening of the two commissures. Commissural splitting was defined as either an increased apparent opening angle of either commissure or increased leaflet separation associated with an increased transverse diameter of the orifice, or both [17, 18].

### Data recorded

We recorded demographic and clinical characteristics for all patients. Venous blood samples were drawn on the day before, and on the day of PMC using EDTA tubes that were centrifuged and stored at − 80 °C for later analysis. Analysis of BNP levels was performed using the ARCHITEXT i2000 system (Abbott Architect System, Abbott Laboratories, Abbott Park, IL, USA); NT-proBNP using the COBAS e601 analyzer (Roche Diagnostics, Indianapolis, IN, USA); MR-proANP was analyzed by immunoluminometric assays (B.R.A.H.M.S. AG, Hennigsdorf, Germany). Finally, sCD146 was measured using the CY-QUANT ELISA sCD146 kit (Biocytex, Marseille, France).

### Statistical analysis

Quantitative data are expressed as mean ± standard deviation when normally distributed, and median [interquartile range] when non-normally distributed. Qualitative data are expressed as number (percentage). Independent variables were compared using the Mann-Whitney U-test, the chi square or Fisher’s exact tests, as appropriate. Paired variables were compared with the paired-samples t-test. Correlations between the relative change in each biomarker, and the relative change in mitral valve area were evaluated using Spearman’s correlation coefficient. A *p*-value < 0.05 was considered statistically significant. All analyses were performed using MedCalc software (MedCalc, Mariakerke, Belgium).

## Results

Forty-eight patients with severe symptomatic MS were identified and screened for inclusion in the study from January 2011 to January 2012. Eight patients (16.6%) had an anatomical form that was incompatible with PMC and were thus excluded. The remaining 40 patients (32 women, 8 men) underwent PMC and constitute the population of this study, and their characteristics are described in Table 1. Thirty patients (75%) had a successful PMC procedure as defined by echocardiographic criteria, and 10 (25%) were considered to have unsuccessful outcome.

**Table 1:** Characteristics of the 40 patients submitted to percutaneous mitral commissurotomy, in the overall population and according to procedural success

	**Whole population (*N*** **= 40)**	**Successful PMC (*n* = 30)**	**Unsuccessful PMC (*n* = 10)**	***p*value**
Age, years	63.5 ± 12.7	60.2 ± 12.8	68.2 ± 10.2	0.08
Female sex	32 (80)	22 (73)	10 (100)	0.6
NYHA class 3–4	23 (57.5)	18 (60)	5 (50)	0.17
Atrial fibrillation	18 (45)	12 (40)	6 (60)	0.46
Previous mitral surgery or PMC	7 (17.5)	4 (13)	7 (70)	0.002
Significant aortic stenosis	6 (15)	5 (17)	1 (10)	0.95
Hypertension	9 (22.5)	5 (17)	4 (40)	0.95
Renal failure (CrCl ≤60 ml/min)	18 (45)	12 (40)	6 (60)	0.46
Obesity	9 (22.5)	7 (23)	2 (20)	0.81
ACEI or ARB	10 (25)	7 (23)	3 (30)	0.98
Beta blockers	20 (50)	13 (43)	7 (70)	0.26
Diuretics	25 (62.5)	17 (57)	8 (80)	0.35
Aldosterone antagonist	8 (20)	4 (13)	4 (40)	0.16
Left atrial diameter, mm	48 ± 7.1	46 ± 7.8	49 ± 6.9	0.28
Left atrial area, cm^2^	32 ± 7.1	32 ± 37	47 ± 6.9	< 0.0001
Left atrial volume, mm^3^	129 ± 42	62 ± 20.5	185 ± 37	< 0.0001
Mitral valve gradient, mmHg	9.1 ± 3.5	9.2 ± 3.5	8.7 ± 3.4	0.7
Mitral valve area, cm^2^	1.1 ± 0.2	1.1 ± 0.2	1.1 ± 0.2	1
LV end-diastolic diameter, mm	49 ± 7.7	49 ± 7.9	46 ± 6.3	0.28
LV end-systolic diameter, mm	31 ± 4.6	31 ± 4.5	30 ± 5	0.55
LVEF %	64 ± 7.9	64 ± 8.1	64 ± 6.9	1
sPAP, mmHg	44 ± 14±	46 ± 15	42 ± 7	0.42
Mitral regurgitation ≥2	8 (20)	6 (20)	2 (20)	0.65
Wilkins score > 8	14 (35)	9 (30)	5 (50)	0.44

*PMC* percutaneous mitral commissurotomy, *NYHA* New York Heart Association, *CrCl* creatinine clearance, *ACEI* angiotension-converting enzyme inhibitor, *ARB* angiotensin-receptor blocker, *LVEF* left ventricular ejection fraction, *sPAP* systolic pulmonary artery pressure

Average age was 63.5 ± 12.7 years; the majority were female (*n* = 32, 80%). Half of the patients were in atrial fibrillation, and almost 60% had New York Heart Association (NYHA) grade 3 or 4 dyspnea.

The echocardiographic findings in the study population are detailed in Table 2. The mean valve area was 1.1 ± 0.2 cm^2^, while mean gradient was 9.0 ± 3.5 mmHg. Median left atrial volume was 129 mm^3^, median left atrial area was 32 cm^2^ and mean systolic pulmonary arterial pressure was 44 ± 14 mmHg. Left ventricular ejection fraction was preserved in all patients. Unfavorable valve anatomy was present in 14 patients (35%) with a Wilkins score > 8; 8 patients (20%) had mitral regurgitation ≥2, and 7 (17.5%) patients had a history of previous PMC or surgical commissurotomy.

**Table 2:** Echocardiographic findings before and after PMC in the overall study population (*n* = 40), and according to procedural success

	**Whole population (*N*** **= 40)**			**Successful PMC (*n*** **= 30)**			**Unsuccessful PMC (*n*** **= 10)**		
	**Pre-PMC**	**Post-PMC**	***p*value**	**Pre-PMC**	**Post-PMC**	***p*value**	**Pre-PMC**	**Post-PMC**	***p*value**
Mitral gradient, mmHg	9.0 ± 3.5	5.1 ± 1.6	< 0.0001	9.3 ± 3.5	4.8 ± 1.5	< 0.0001	8.7 ± 3.6	6.1 ± 1.6	0.06
Mitral valve area, cm^2^	1.1 ± 0.2	1.7 ± 0.3	< 0.0001	1.1 ± 0.2	1.8 ± 0.3	< 0.0001	1.1 ± 0.3	1.3 ± 0.1	0.06
LVEF, %	63 ± 7.9	62 ± 14.7	0.8	64 ± 8.1	66 ± 11.2	0.43	64 ± 7.3	53 ± 18.2	0.09
sPAP, mmHg	44 ± 14	36 ± 8	0.0003	46 ± 15	36 ± 9	0.003	42 ± 7	34 ± 6	0.01

*PMC* percutaneous mitral commissurotomy, *LVEF* left ventricular ejection fraction, *sPAP* systolic pulmonary artery pressure

*P*-values are for the comparison before vs after by paired Student t test

PMC yielded an average increase of 0.6 cm^2^ in planimetric mitral valve area (0.7 cm^2^ in those with successful procedure (*n* = 30) vs. 0.2 cm^2^ in those with unsuccessful procedure (*n* = 10)) and an average reduction of 3.9 mmHg in mitral gradient (4.5 mmHg in those with successful procedure vs. 2.6 mmHg in those with unsuccessful procedure, respectively). Systolic pulmonary artery pressure was significantly reduced after PMC (− 8 mmHg, *p* = 0.0003) (Table 2).

Patients with unsuccessful procedure were on average slightly older, with significantly more frequent history of PMC or mitral surgery and larger left atria despite similar grade of MS than those in whom the procedure was successful (Table 1), perhaps indicating a longer history of disease.

**Fig. 1:**
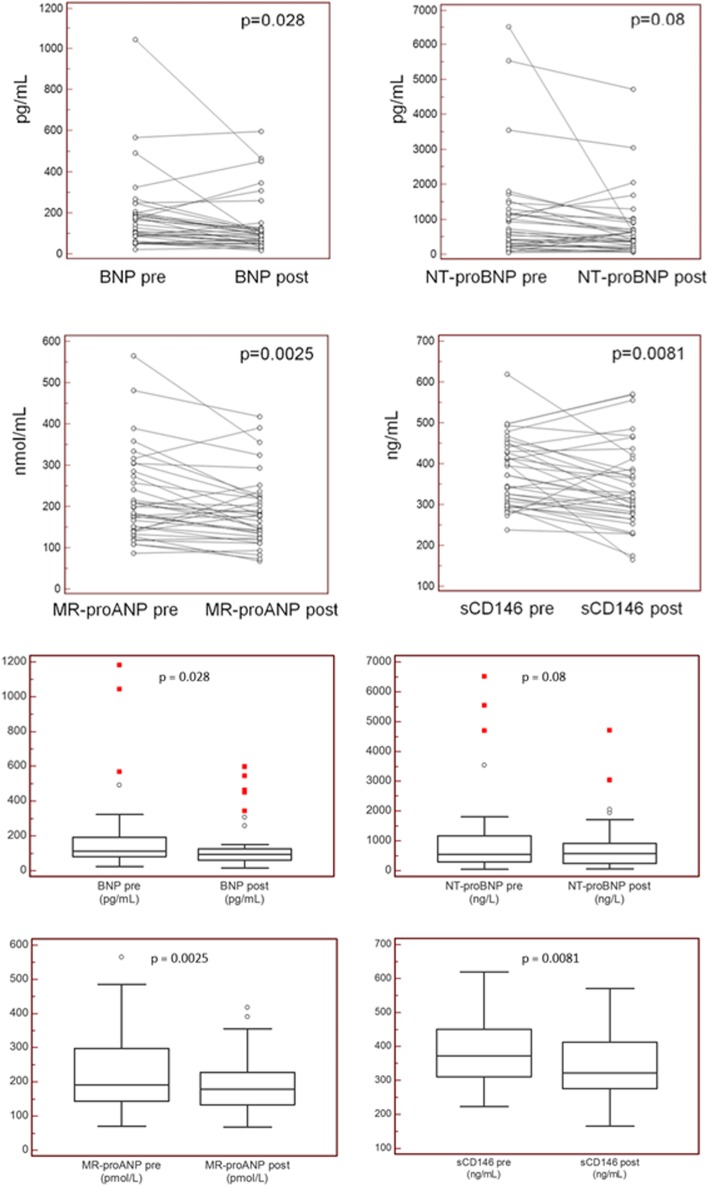
Individual data for the plasma cardiovascular biomarkers under study, before (pre) and after (post) percutaneous mitral commissurotomy for each patient (top panels) and overall (bottom panels)

**Fig. 2:**
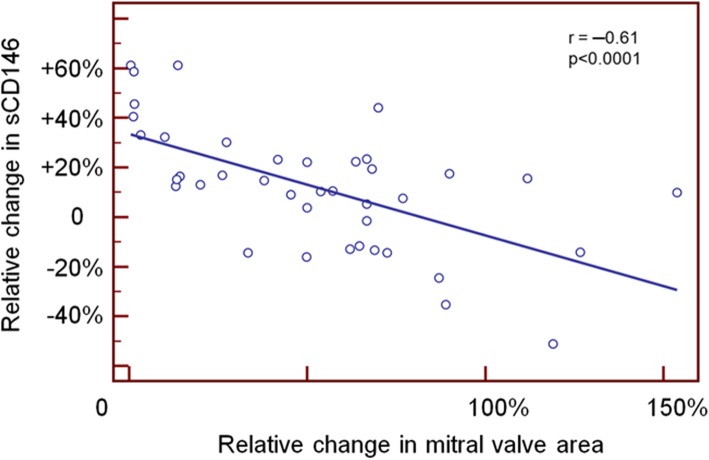
Correlation between relative change in sCD146 and relative change in mitral valve area

Circulating cardiovascular biomarkers before and after PMC are shown in Table 3 and Fig. 1. Before PMC, MR-proANP and sCD146 were markedly elevated in the large majority of patients with severe MS. In the overall population, PMC yielded a significant reduction in plasma MR-proANP and sCD146. Significant reductions in these two biomarkers were seen in patients with successful procedural outcome, whereas no significant reduction was observed in MR-proANP and sCD146 in patients who had an unsuccessful procedure (Table 3). There was no significant difference between pre-and post-PMC levels of NT proBNP. Conversely, BNP, MR proANP and sCD146 were all significantly lower after PMC, compared to pre-PMC levels. Notably, a significant correlation was found between changes in plasma sCD146 and the relative change in mitral valve area (Fig. 2).

**Table 3:** Plasma levels of the 4 cardiovascular biomarkers under study before and after PMC, in the overall population and according to procedural success

	**Whole population (*N* = 40)**			**Successful PMC (*n* = 30)**			**Unsuccessful PMC (*n* = 10)**		
	**Pre-PMC**	**Post-PMC**	***p*value**	**Pre-PMC**	**Post-PMC**	***p*value**	**Pre-PMC**	**Post-PMC**	***p*value**
BNP, pg/mL	182 [80–194]	132 [56–125]	0.028	184 [63–194]	122 [53–123]	0.03	176 [87–193]	161 [60–344]	0.62
NT-proBNP, pg/mL	1050[232–1171]	739 [219–909]	0.08	1067 [200–1171]	654 [141–706]	0.08	999 [311–1128]	994 [375–1951]	0.97
MR-proANP, nmol/mL	220 [142–273]	187 [130–224]	0.0025	219 [142–257]	179 [123–219]	0.0012	223 [139–284]	210 [133–347]	0.6
sCD146, ng/mL	379 [308–441]	343[277–416]	0.0081	370 [298–427]	327 [268–378]	0.002	406 [323–483]	389 [283–555]	0.7

*PMC* percutaneous mitral commissurotomy. *P*-values are for the comparison before vs after by paired Student t test

Reference values for middle-aged women: BNP < 100 pg/ml, NT-proBNP< 300 pg/mL, MR-proANP< 85 nmol/mL, sCD146 still under investigation, likely < 320 ng/mL

**Fig. 3:**
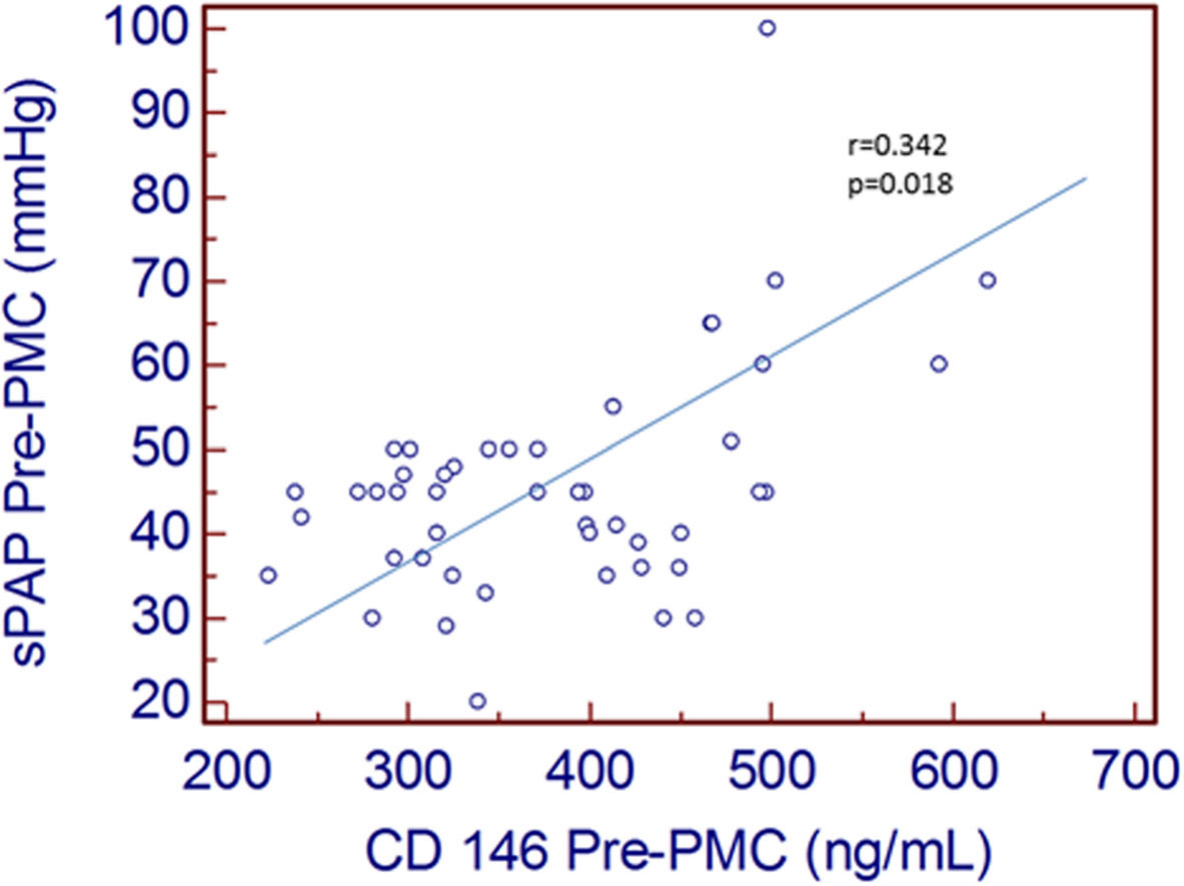
Correlation between sPAP pre-PMC and sCD146 pre-PMC

**Fig. 4:**
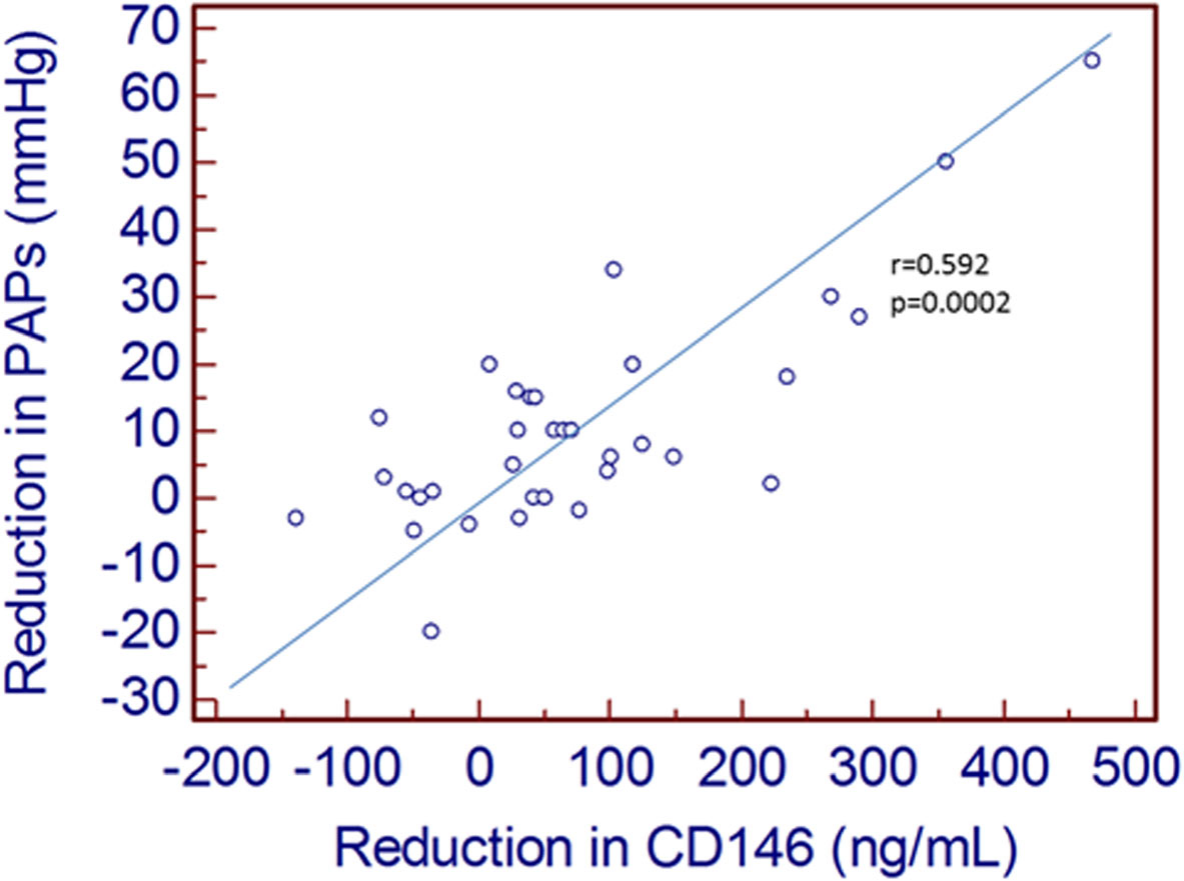
Correlation between reduction in sPAP and reduction in sCD146. Negative values mean increase of sCD146 or sPAP between pre and post-PMC

Elevated pre-procedural sPAP correlated with high sCD146 (Fig. 3), but not MR-proANP. Accordingly, a significant correlation between the decrease in sPAP and sCD146 after PMC was shown (Fig. 4), whereas no correlation between changes in sPAP and MR-proANP was shown.

## Discussion

Our study describes the behaviour of cardiovascular biomarkers before and after PMC in patients with severe MS and investigated the potential role of those cardiovascular biomarkers to assess procedural success of PMC. We showed a significant decrease in plasma levels of MR-proANP and sCD146 after successful PMC, whereas no such decrease in these biomarkers is observed in patients in whom the procedure is not successful.

ANP is a natriuretic peptide specific to the atria, and a sandwich immunoassay has been developed specifically for the assessment of MR-proANP [19], which is more stable and a more reliable analyte for measurement than mature ANP and its C- and N-terminal segments [20, 21]. The link between MR-proANP and congestion has previously been established [7], but to date, there has been little research into the utility of MR-proANP in pathologies specifically affecting the left atrium. In this regard, our study provides further evidence that patients with elevated LA pressure due to MS and preserved LVEF have elevated MR-proANP levels, which decline after successful PMC, without any significant relation with the variation in pulmonary arterial pressure. We can therefore hypothesize that the elevated left atrial pressure induces wall stretch, leading to release of MR-proANP and resulting in elevated plasma concentrations. Successful PMC leads to an improvement in hemodynamics, with a drop in left atrial pressure, and thus, a corresponding drop in plasma levels of MR-proANP. Our findings are coherent with earlier publications showing a reduction in ANP after PMC, and a correlation between changes in plasma ANP and those in left atrial pressure [22]. Furthermore, MR-proANP has been shown to be a predictor of adverse cardiac events after percutaneous repair of severe mitral valve regurgitation [8]. Therefore, it would seem that MR-proANP is a marker of left atrial disease.

Previous publications have reported that sCD146 was useful for identifying signs of left heart failure, and the cardiac origin of dyspnea [10, 11]. However, the pathophysiology of sCD146 release is not well understood in the context of pulmonary congestion without left-ventricular failure. In our study, all patients had preserved LVEF, and there was no evidence of increased left ventricular pressure. Therefore, the elevated pre-procedural sCD146 concentrations are unlikely to be related to left ventricular release. Our results plead more in favour of the hypothesis that sCD146 is released at least partially by the pulmonary vasculature. MS leads to pulmonary congestion, which is reduced by successful PMC, providing a potential explanation for the reduction in sCD146 after successful procedure (PMC), and for the link between pulmonary artery pressure and sCD146. This hypothesis is in line with previously published data showing that sCD146 is markedly elevated in patients with pulmonary hypertension, and that it is particularly elevated in patients with MS when their pulmonary artery pressure exceeds 30 mmHg [23]. sCD146 has also been shown to reflect systemic congestion [12]. Unsuccessful PMC only yields a small reduction in systolic PAP that has little or no effect on right ventricular hemodynamics. Consequently, no improvement in systemic congestion ensues, thus explaining why there is no significant reduction in sCD146 after unsuccessful PMC.

It is established that sCD146 is at least partially released by the endothelium, and this fits with our hypothesis [9]. Taken together, these data indicate that sCD146 could be a relevant marker of PMC success by reflecting the hemodynamic improvement achieved by the successful procedure (i.e. reduction in pulmonary and systemic congestion). They also suggest that sCD146 could be a marker of pulmonary congestion, released in the presence of increased pulmonary pressure, or a marker of systemic congestion, in line with previous publications [10–12].

The success of PMC is usually evaluated by transthoracic echocardiography (TTE). However, this technique suffers from several limitations, including the echogenicity of the patient, intra-operator variability, operator experience, and variability linked to changes in the loading conditions in a same patient. In this context, assessment of biomarkers that reflect immediate hemodynamic improvement seems to be a good indicator of PMC success, and less subject to variability than TTE. Data are controversial regarding the utility of more widely used biomarkers, such as BNP and NT-proBNP, to assess the success of PMC, precluding their use as a reliable indicator in this context [22, 24–26]. Our results suggest that the drop in sCD146 and MR-proANP could indicate immediate procedural success after PMC, reflecting the hemodynamic improvement achieved by the procedure, especially since the magnitude of the drop in sCD146 is related to the benefit yielded by the PMC. To the best of our knowledge, this is the first study to report the utility of assessing sCD146 and MR-proANP in the context of PMC.

Our study nonetheless has some limitations that deserve to be underlined. Firstly, the sample size is relatively small, although comparable to that of other studies in the field. Clearly, the sample size is too small to allow identification of a threshold for procedural success for these two biomarkers, and consequently, also too low to allow calculation of their sensitivity and specificity. Furthermore, we also lack additional hemodynamic parameters, notably invasively measured left atrial pressure measurements. Finally, our data are from the immediate post-procedural period, and no follow-up data regarding clinical, echographic or biological parameters were available in the medium to long term. Therefore, further studies are warranted to elucidate the pathophysiological mechanisms underpinning the changes in these biomarkers and to identify their exact relevance in this indication.

## Conclusion

MR-proANP decreased significantly after PMC, likely due to a reduction in atrial stretch achieved by the successful procedure. Plasma sCD146 decreased significantly immediately after successful PMC, likely due to reduced endothelial release secondary to a reduction in pulmonary pressure. MR-proANP and sCD146 appear to be markers of immediate success of PMC and of the hemodynamic improvement achieved by this procedure in patients with MS.

## Data Availability

The datasets used and/or analysed during the current study are available from the corresponding author on reasonable request.

## Abbreviations

ANP: Atrial natriuretic peptide
BNP: Brain natriuretic peptide
LA: Left atrium/atrial
LVEF: Left ventricular ejection fraction
MRproANP: Mid-regional pro-atrial natriuretic peptide
MS: Mitral stenosis
NTproBNP: N-terminal pro-B-type natriuretic peptide
PMC: Percutaneous mitral commissurotomy
sCD146: Soluble CD146
sPAP: Systolic pulmonary artery pressure
TOE: Trans-oesophageal echocardiography
TTE: Transthoracic echocardiography

## References

[1] Ponikowski P, Voors AA, Anker SD, Bueno H, Cleland JG, Coats AJ, Falk V, Gonzalez-Juanatey JR, Harjola VP, Jankowska EA (2016). ESC guidelines for the diagnosis and treatment of acute and chronic heart failure: the task force for the diagnosis and treatment of acute and chronic heart failure of the European Society of Cardiology (ESC). Developed with the special contribution of the heart failure association (HFA) of the ESC. Eur J Heart Fail.

[2] Yancy CW, Jessup M, Bozkurt B, Butler J, Casey DE, Drazner MH, Fonarow GC, Geraci SA, Horwich T, Januzzi JL (2013). ACCF/AHA guideline for the management of heart failure: a report of the American College of Cardiology Foundation/American Heart Association task force on practice guidelines. J Am Coll Cardiol.

[3] Troughton R, Michael Felker G, Januzzi JL (2014). Natriuretic peptide-guided heart failure management. Eur Heart J.

[4] Harada E, Mizuno Y, Kugimiya F, Shono M, Maeda H, Yano N, Kuwahara K, Yasue H (2017). B-type natriuretic peptide in heart failure with preserved ejection fraction- relevance to age-related left ventricular modeling in Japanese. Circ J.

[5] Khalid U, Wruck LM, Quibrera PM, Bozkurt B, Nambi V, Virani SS, Jneid H, Agarwal S, Chang PP, Loehr L (2017). BNP and obesity in acute decompensated heart failure with preserved vs. reduced ejection fraction: the atherosclerosis risk in communities surveillance study. Int J Cardiol.

[6] Meijers WC, Hoekstra T, Jaarsma T, van Veldhuisen DJ, de Boer RA (2016). Patients with heart failure with preserved ejection fraction and low levels of natriuretic peptides. Neth Heart J.

[7] Van Aelst LNL, Arrigo M, Placido R, Akiyama E, Girerd N, Zannad F, Manivet P, Rossignol P, Badoz M, Sadoune M (2018). Acutely decompensated heart failure with preserved and reduced ejection fraction present with comparable haemodynamic congestion. Eur J Heart Fail.

[8] Wohrle J, Karakas M, Trepte U, Seeger J, Gonska B, Koenig W, Rottbauer W (2015). Midregional-proAtrial natriuretic peptide and high sensitive troponin T strongly predict adverse outcome in patients undergoing percutaneous repair of mitral valve regurgitation. PLoS One.

[9] Boneberg EM, Illges H, Legler DF, Furstenberger G (2009). Soluble CD146 is generated by ectodomain shedding of membrane CD146 in a calcium-induced, matrix metalloprotease-dependent process. Microvasc Res.

[10] Gayat E, Caillard A, Laribi S, Mueller C, Sadoune M, Seronde MF, Maisel A, Bartunek J, Vanderheyden M, Desutter J (2015). Soluble CD146, a new endothelial biomarker of acutely decompensated heart failure. Int J Cardiol.

[11] Kubena P, Arrigo M, Parenica J, Gayat E, Sadoune M, Ganovska E, Pavlusova M, Littnerova S, Spinar J, Mebazaa A (2016). Plasma levels of soluble CD146 reflect the severity of pulmonary congestion better than brain natriuretic peptide in acute coronary syndrome. Ann Lab Med.

[12] Arrigo M, Truong QA, Onat D, Szymonifka J, Gayat E, Tolppanen H, Sadoune M, Demmer RT, Wong KY, Launay JM (2017). Soluble CD146 is a novel marker of systemic congestion in heart failure patients: an experimental mechanistic and Transcardiac clinical study. Clin Chem.

[13] Wilkins GT, Weyman AE, Abascal VM, Block PC, Palacios IF (1988). Percutaneous balloon dilatation of the mitral valve: an analysis of echocardiographic variables related to outcome and the mechanism of dilatation. Br Heart J.

[14] Helmcke F, Nanda NC, Hsiung MC, Soto B, Adey CK, Goyal RG, Gatewood RP (1987). Color Doppler assessment of mitral regurgitation with orthogonal planes. Circulation.

[15] Frea S, Capriolo M, Marra WG, Cannillo M, Fusaro E, Libertucci D, Morello M, Gaita F (2011). Echo Doppler predictors of pulmonary artery hypertension in patients with systemic sclerosis. Echocardiography.

[16] Tuzcu EM, Block PC, Griffin BP, Newell JB, Palacios IF (1992). Immediate and long-term outcome of percutaneous mitral valvotomy in patients 65 years and older. Circulation.

[17] Fatkin D, Roy P, Morgan JJ, Feneley MP (1993). Percutaneous balloon mitral valvotomy with the Inoue single-balloon catheter: commissural morphology as a determinant of outcome. J Am Coll Cardiol.

[18] Reid CL, McKay CR, Chandraratna PA, Kawanishi DT, Rahimtoola SH (1987). Mechanisms of increase in mitral valve area and influence of anatomic features in double-balloon, catheter balloon valvuloplasty in adults with rheumatic mitral stenosis: a Doppler and two-dimensional echocardiographic study. Circulation.

[19] Morgenthaler NG, Struck J, Thomas B, Bergmann A (2004). Immunoluminometric assay for the midregion of pro-atrial natriuretic peptide in human plasma. Clin Chem.

[20] Ala-Kopsala M, Magga J, Peuhkurinen K, Leipala J, Ruskoaho H, Leppaluoto J, Vuolteenaho O (2004). Molecular heterogeneity has a major impact on the measurement of circulating N-terminal fragments of A- and B-type natriuretic peptides. Clin Chem.

[21] Seidler T, Pemberton C, Yandle T, Espiner E, Nicholls G, Richards M (1999). The amino terminal regions of proBNP and proANP oligomerise through leucine zipper-like coiled-coil motifs. Biochem Biophys Res Commun.

[22] Nakamura M, Kawata Y, Yoshida H, Arakawa N, Koeda T, Ichikawa T, Funakoshi T, Hiramori K (1992). Relationship between plasma atrial and brain natriuretic peptide concentration and hemodynamic parameters during percutaneous transvenous mitral valvulotomy in patients with mitral stenosis. Am Heart J.

[23] Badoz M, Arrigo M, Iung B, Amioglu G, Yilmaz MB, Meneveau N, Sadoune M, Brunette A, Mebazaa A, Seronde MF (2016). Role of cardiovascular biomarkers for the assessment of mitral stenosis and its complications. Eur J Intern Med.

[24] Esteves WA, Lodi-Junqueira L, Neto CP, Tan TC, Nascimento BR, Mehrotra P, Barbosa MM, Ribeiro AL, Nunes MC (2013). The impact of right ventricular stroke work on B-type natriuretic peptide levels in patients with mitral stenosis undergoing percutaneous mitral valvuloplasty. J Interv Cardiol.

[25] Pourafkari L, Seyedhosseini S, Kazemi B, Esmaili H, Aslanabadi N (2014). Changes in serum NT-pro BNP and left atrial BNP levels after percutaneous Transvenous mitral Commissurotomy in sinus rhythm versus atrial Firilation. J Cardiovasc Thorac Res.

[26] Ramakrishnan S, Agarwal A, Singh S, Karthikeyan G, Seth S, Narang R, Bhargava B (2010). NT-pro-BNP levels as a marker of success of percutaneous transvenous mitral commissurotomy. Indian Heart J.

